# The Microgeographical Patterns of Morphological and Molecular Variation of a Mixed Ploidy Population in the Species Complex *Actinidia chinensis*

**DOI:** 10.1371/journal.pone.0117596

**Published:** 2015-02-06

**Authors:** Yifei Liu, Dawei Li, Ling Yan, Hongwen Huang

**Affiliations:** 1 Key Laboratory of Plant Resources Conservation and Sustainable Utilization, South China Botanical Garden, the Chinese Academy of Sciences, Guangzhou, Guangdong, P. R. China; 2 Key Laboratory of Plant Germplasm Enhancement and Specially Agriculture, Wuhan Botanical Garden, the Chinese Academy of Sciences, Wuhan, Hubei, P. R. China; University of Massachusetts, UNITED STATES

## Abstract

Polyploidy and hybridization are thought to have significant impacts on both the evolution and diversification of the genus *Actinidia*, but the structure and patterns of morphology and molecular diversity relating to ploidy variation of wild *Actinidia* plants remain much less understood. Here, we examine the distribution of morphological variation and ploidy levels along geographic and environmental variables of a large mixed-ploidy population of the *A. chinensis* species complex. We then characterize the extent of both genetic and epigenetic diversity and differentiation exhibited between individuals of different ploidy levels. Our results showed that while there are three ploidy levels in this population, hexaploids were constituted the majority (70.3%). Individuals with different ploidy levels were microgeographically structured in relation to elevation and extent of niche disturbance. The morphological characters examined revealed clear difference between diploids and hexaploids, however tetraploids exhibited intermediate forms. Both genetic and epigenetic diversity were high but the differentiation among cytotypes was weak, suggesting extensive gene flow and/or shared ancestral variation occurred in this population even across ploidy levels. Epigenetic variation was clearly correlated with changes in altitudes, a trend of continuous genetic variation and gradual increase of epigenomic heterogeneities of individuals was also observed. Our results show that complex interactions between the locally microgeographical environment, ploidy and gene flow impact *A. chinensis* genetic and epigenetic variation. We posit that an increase in ploidy does not broaden the species habitat range, but rather permits *A. chinensis* adaptation to specific niches.

## Introduction

Polyploidy and hybridization are important processes involved in plant diversification and speciation [1, 2]. Polyploidization *per se* can generate duplicated genomes, and subsequent evolutionary changes can result in dynamic alterations in structural and functional variations of the duplicated genomes at both genetic and epigenetic levels [2, 3, 4, 5]. The incidence of polyploidizations may have important phenotypic and ecological consequences such as increasing the extent of trait variation and/or capacity for adaptation to novel niches [6, 7, 8].

In plants, the coexistence of different ploidy levels is common [9, 10, 11, 12], making them ideal systems for studying the evolutionary mechanisms and processes involving in the origin and maintenance of sympatric polyploid complexes. At a broad spatial scale, the stable or transient sympatric coexistence of different ploidy levels has been explained by both balancing and directional selection [13]. At smaller spatial scales, the coexistence of multiple ploidy levels is thought to be due to partial niche differentiation [6]. Polyploid lineages persisting in the presence of natural hybridization or gene flow across ploidy suggests that the cross-talk between genetic and ecological factors in relation to polyploid evolution is complicated [11]. Different ploidy individuals can vary significantly in their response to hybridization, polyploidy or the combination of the two along environmental variables [8].

Studying the patterns of genetic diversity and differentiation throughout the whole genomes of individuals varying in ploidy level within a species complex can assist in our understanding on its origin and evolution. In plants, the potential for genetic exchange across ploidy levels has long been recognized [14], but it has rarely been observed in the wild, particularly at a microgeographical level [11, 15]. Reproductive isolation presented between individuals of different ploidy levels can lead to cryptic speciation [16]. However, substantial gene flow among cytotypes can also have significant impacts on both the morphology and ecology of polyploids [17].

Epigenetic information encoded by variation in DNA methylation and chromatin marking, is involved into gene regulation, chromosome function and adaptation in plants [18]. DNA methylation is the best-described epigenetic mechanism in plants: CG, CHG and CHH (H stands for A, C or T) may all be subject to methylation, though CG methylation is considered to be the most stable [18]. In non-model organisms, investigations into the level and pattern of genome-wide DNA methylation is a first step towards assessing the potential significance of epigenotypes to phenotypic traits and ecological adaptation [18, 19]. In natural populations of plants, however, elucidating the effects of polyploidy and hybridization on both the genetic and epigenetic interactions underlying adaptive traits is difficult despite natural epialleles have started to be identified [18, 20].

**Fig 1:**
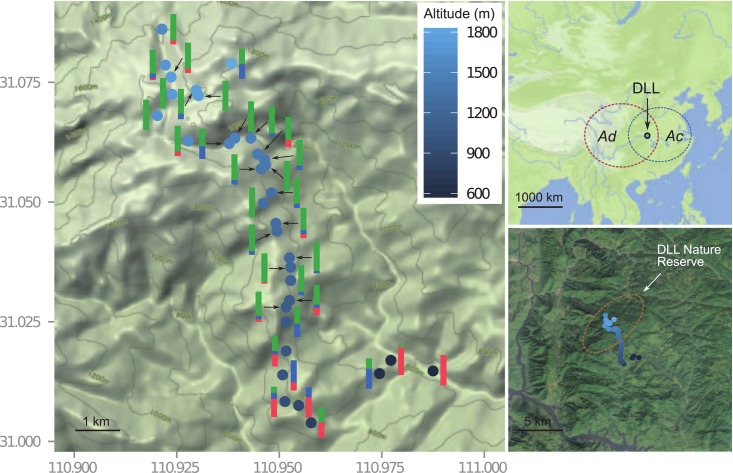
Sampling scheme of *Actinidia chinensis* complex in the Dalaoling (DLL) population. The vertical bar with different color composition shows the variation of ploidy levels in each sampling site (diploid: pink; tetraploid: sky blue; hexaploid: green). The top-right small map shows the location of DLL population in China, while both dashed-line ellipses show the potentially geographical distribution of both varieties *Ac* and *Ad*. The bottom-right map shows the DLL Nature Reserve. Details on the population and frequency of ploidy individuals are given in S1 Table. The map base was generated using free data sources from the National Geomatics Center of China.

Kiwifruit (*Actinidia* Lindl.) is an important horticultural fruit tree crops, and is widely planted around the world. The genus *Actinidia* comprises about 54 species [21]. All *Actinidia* species are perennial and dioecious plants with a climbing or straggling growth habit. Commercial cultivars of kiwifruit are mostly derived from the species complex *A*. *chinensis* Planch., which includes two main varieties *A*. *chinensis* var. *chinensis* (hereafter *Ac*) and *A*. *chinensis* var. *deliciosa* (*Ad*). The morphological delimitation of the two varieties is based on characteristics such as the hair types covering on flowering branchlets and fruits (*Ac*: finely tomentose; *Ad*: brown strigose hairs), the presence of over-wintering buds (*Ac*: exposed; *Ad*: buried in the bark) and the matured fruit flesh color (*Ac*: mostly yellow or yellow-green; *Ad*: mostly green). However, morphological intermediates of both varieties are widely recognized, in particular in the region in central and western China where their ranges overlap [22, 23, 24]. At a broad geographical-scale, *Ac* grows better in lowlands than *Ad* in eastern and central China, while *Ad* is more tolerant to the alpine habitats that dominate in central and western China (Fig. 1)[25, 26]. Cytogenetic analysis has showed that *A*. *chinensis* ploidy levels vary from diploid to hexaploid (2*n* = 2*x* = 58, *Ac*: 2*x* and 4*x*, *Ad*: 6*x* with few 4*x*)[23, 27]. Recurrent whole genome duplications and/or hybridization in the genus *Actinidia* have been invoked to explain the diversification of kiwifruit [28, 29]. Populations with mixed ploidy levels have been detected in regions of co-occurrence of both varieties [23] and ploidy levels are, to some degree, correlated with geographical distribution and morphological variation across large geographical regions [23, 30].

Due to the economic importance of kiwifruit, most studies of *A*. *chinensis* focus on breeding and evaluations of the horticultural merits of specific germplasm [26, 31]. The population structure of wild *A*. *chinensis* with mixed ploidy levels has received less attention, particularly at a small or microgeographical scale. In this study, we selected a population of the *A*. *chinensis* complex with representative ploidy diversity to examine morphological and molecular diversity of kiwifruit at a fine spatial scale along a continuous sampling transect. We first analyzed geo-environmental variables, morphological characters and ploidy levels. We then used amplified fragment length polymorphism (AFLP) markers [32] and methylation sensitive amplified polymorphisms (MSAP) [33] to assess the genetic and epigenetic differentiation relating to genome duplication and hybridization. We specifically addressed whether ploidy levels, morphological variation, and genetic and epigenetic diversity of individuals microgeographical structured, and whether individuals varying in ploidy levels reflect genetic/epigenetic subdivisions or are interconnected by extensive gene flow at fine spatial scale. All these information will help us understand the mechanism driving the evolution and diversification of polyploid kiwifruit.

## Materials and Methods

### Study area and sampling procedure

The study area is the Dalaoling (DLL) mountain (30°52′~31°07′N, 110°51′~111°00′E) of Yichang, Hubei, China (Fig. 1). The DLL mountain is a subset of the eastern part of the Daba Mountains in the Three Gorges region, and has a highly variable montane topology and diverse ecological conditions. Different soil types are found in the altitudinal ranges between 800–1600 m a.s.l., while mountain brown soil dominate above 1600 m a.s.l. The flora of the DLL mountain is predominantly temperate, with a high endemism in Eastern-Asian and Chinese elements, but also exhibits some historical connections with tropical flora. Significant anthropogenic disturbances are rare above 800 m a.s.l. due to the protection as a natural reserve by the local government.

Thirty-four sampling sites along an altitudinal gradient (500 ~ 2000 m a.s.l.) were selected in the DLL mountain area between 2008 and 2009 with the permission of the managing office of the Dalaoling natural reserve (Fig. 1). The sites were approximately equidistant and each site had at least one variety of *Ac* or *Ad*. At least two individuals were sampled at each site, producing a total of 272 individuals across the transect (S1 Table). Sixteen morphological characteristics based on the International Union for the Protection of New Varieties of Plants (UPOV) descriptors (*A*. *chinensis*, http://www.upov.int/genie/en/details.jsp?id=101) were used to record the morphology of samples in the field in August of 2010 (S2 Table). Characteristics 1–6 (see details in S2 Table) are quantitative and recorded as means of the measurements (four measurements per plant), and characteristics 7–16 are qualitative and coded as different states (0, 1, 2, etc.). Only adult plants with similar diameter at breast height (DBH, 3–5 cm) were analyzed (263 out of 272) and juvenile plants (1~3 years old plants that have never flowered) were excluded. For each site, geographical and environmental variables were measured and recorded in a 10 m ×10 m ecological relevé, including GPS coordinates, slope steepness (°), aspect, mesotopography, soil type, niche disturbance and vegetation cover (S3 Table).

### Estimate of ploidy levels

The ploidy level of each individual was determined by a flow cytometric measurement (FCM) using a CyFlow Ploidy Analyser (Partec, Germany). The FCM is based on the comparison between the relative fluorescence of the sample and that of a standard. The detailed FCM method was performed as in Li *et al*. [23], and a kiwifruit cultivar ‘Hongyang’ derived from *Ac* (2*n* = 2*x* = 58), whose chromosome number had been determined previously based on chromosome counts and the estimated genome size of 750 Mb [31], was used as an internal reference standard.

### AFLP and MSAP genotyping

A representative subset of 192 individuals (at least one from each site) was further used for genome-wide genetic and epigenetic analyses (S1 Table). Total genomic DNA was extracted from dried leaf material according to a CTAB protocol [34] with minor modifications.

The AFLP procedure was performed according to a standard method [32]. Approximately 500 ng of genomic DNA were digested with both *Mse*I and *EcoR*I commonly (10 U for each, New England Biolabs Inc., Beverly, MA, USA) at 37°C overnight, and then ligated with adaptors using 4 U T4 DNA ligase (Promega) at 16°C for at least 4 h. The restriction-ligation products were subsequently diluted tenfold for pre-amplification PCR. A total reaction volume of 15 μl was used containing 10× PCR Buffer, 2 mM MgCl_2_, 1 mM dNTP mix, 0.5 μM each *EcoR*I-A and *Mse*I-C primers and 0.25 U DNA Polymerase (Fermentas, Vilnius, Lithuania). The thermal profile used was initial denaturation for 3 min at 94°C, followed by 20 cycles of 94°C for 20 s, 56°C for 30 s, and 72°C for 2 min with a final 30 s extension at 72°C. The tenfold diluted pre-selective PCR product was used as template for selective PCR reactions with the same reaction volume by using fluorescence labeled selective primers. The reaction profile was kept at 94°C for 3 min, followed by 13 cycles of 94°C for 30 s, a touchdown annealing temperature (from 65°C to 56°C, decreasing 0.7°C per cycle) for 1 min to improve amplification, and 72°C for 1 min, and then 23 cycles of 94°C for 30 s, 56°C for 1 min, and 72°C for 1 min with a final 8 min extension at 72°C. The selective PCR products were electrophoresed on an ABI 3730 XL DNA sequencer and dominant fragments were sized using GeneMapper 3.7 (Applied Biosystems, USA). Representative individuals from each ploidy level were genotyped twice on separate lanes to check for possible genotyping error and only fragements (50–500bp) presented consistently across samples with signals above a threshold value recommended by the manufacturer were used for data collection. Internal size standards were used in each lane for exact calibration of different individuals against each other.

MSAP is a modified version of standard AFLP using a pair of methylation-sensitive restriction enzymes *Hpa*II and *Msp*I replacing *Mse*I. Both enzymes are isoschisomers recognizing the same tetranucleotide 5′-CCGG but have differential sensitivity to methylation at the inner or outer cytosine [33]. Methylated CpG are restricted by *Msp*I only, and hemimethylated CpCpG sites are restricted by *Hpa*II only. Sites that are hypermethylated (i.e., both at the internal and external Cs), and sites that are fully methylated at the external Cs (i.e. on both strands) are not cut by either enzymes, whereas sites that are free from methylation are restricted by both [35]. All reactions and genotyping patterns are performed in the same way as the AFLP procedure. All adaptors and primers used for AFLP and MSAP are listed in S4 and S5 Tables.

### Statistical analyses

All statistical analyses were run in R [36] unless mentioned otherwise. Morphological variation among the 263 analyzed individuals was summarized using principal components analysis (PCA). Data were standardized in which all variables were scaled to have unit variance and further were centred by subtracting the mean of a variable before PCA. To test if variation in morphology could be explained by difference in ploidy level, we used a one-way analysis of variance (ANOVA) with ploidy as factor and each principal component (PC) from the PCA of morphological variation as response variable. Post-hoc Tukey HSD tests were used for pairwise comparison of the significance among ploidy levels. Linear discriminant analysis was used to test whether individuals could be correctly reassigned to their derived cytotypes based on principal components of morphology. The respective contribution of each environmental variable to spatial distribution of cytotypes was analyzed by an ordination method of canonical correspondence analysis (CCA). The significance tests of both the total and the respective contributions of the nine constrained variables were conducted with 999 permutations.

For both genetic and epigenetic analyses, only clear and reproducible bands were scored. The AFLP genotyping profile was transformed into a binary matrix representing fragment presence (1) and absence (0) of each locus for further analyses. The method of Herrera and Bazaga [35] was used to code the MSAP profile. Briefly, for each individual a band present in both *Msp*I and *Hpa*II profiles is classified as a nonmethylated site, while a band present in either *Msp*I or *Hpa*II profile is classified as a methylated state. If a band is absent from both enzyme profiles, it is treated as a missing score because it is uninformative since both fragment absence and hypermethylation are possible. Individual fragments were then classified as ‘methylation-susceptible’ when the observed proportion of discordant *Msp*I-*Hpa*II scores exceeded a 5% threshold. Otherwise, it was classified as ‘nonmethylated’. Nonmethylated loci were coded as in AFLP, and instances of discordant *Msp*I-*Hpa*II scores in nonmethylated sites were resolved as fragment presences. Methylation-susceptible fragments were scored as if the methylated state were an imperfectly assessed dominant marker: 1 for the methylated state, 0 for the nonmethylated state and unknown (i.e. score missing) for uninformative state.

Band-based strategies [37] were adopted for the statistical analyses of both AFLP and MSAP results. The amount of genetic and epigenetic diversity for individuals in each ploidy level was assessed by the Shannon diversity index (*S*) using the formula *S* = -∑*P*_*i*_log_2_(*P*_*i*_), in which *P*_*i*_ is the frequency of the band presence at the *i*th locus within population. Phenotypic variation of AFLP and MSAP band profiles was summarized with PCA analyses. One-way ANOVA using ploidy as factor on PCs was carried out to test for overall significance and post-hoc Tukey HSD tests for pairwise significance analyses. Multilocus genetic and epigenetic differentiation of individuals with different ploidy levels was further tested using analyses of molecular variance (AMOVA). To investigate the possible relationship between molecular variation and morphology, the main PCs in both morphological and molecular (genetic and epigenetic) PCA analyses were used to calculate distance matrices for carrying out Mantel tests [38]. Two environmental factors ALT and DIS, which were positively related to morphological variation (see results), were further used as dependent variables to do multiple linear regressions against the PC scores at both genetic and epigenetic levels. Finally, population genetic and epigenetic structure were detected by using STRUCTURE v2.2 [39] using a recessive allele model. No prior information such as ploidy levels of individuals was used and a burn-in period of 50,000 iterations and a sampling period of 100,000 iterations were run on each *K* value from 1 to 10. The optimal *K* values of both data were determined by the increase of the posterior probability of the data (PPD) using the formula *∆K* = Ln PPD_*k*_—Ln PPD_*k*-1_ [40].

## Results

### Variation in ploidy, morphology and ecology

**Fig 2:**
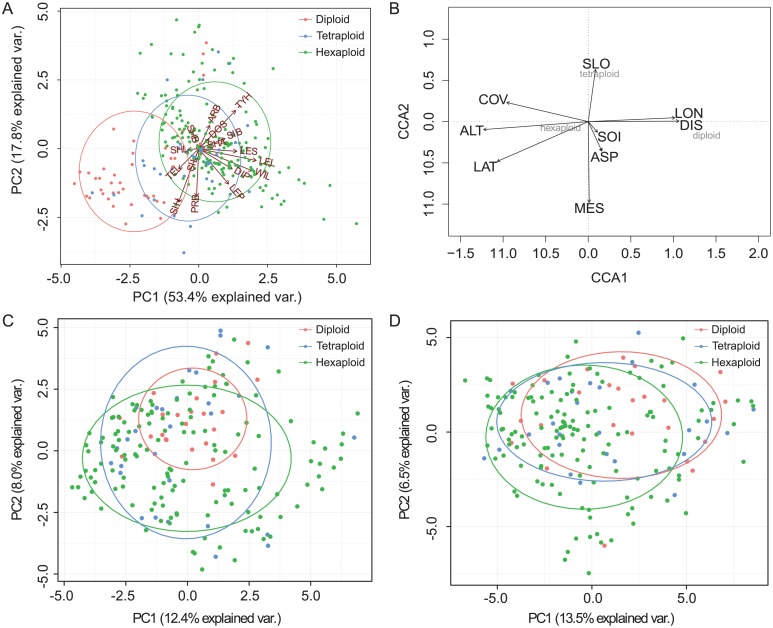
Principal components analysis (PCA) for morphological (a) and molecular variation (c: genetic; d: epigenetic), and (b) canonical correspondence analysis (CCA) for environmental variables and cytotype distribution of *Actinidia chinensis*. PCAs (a, c and d) only present the first two principal components and ellipses represent the dispersion of those points around their center. Abbreviations in PCA analysis of morphological variation are given in S2 Table and abbreviations in CCA analysis are given in Table 1.

FCM analyses mostly produced high-resolution histograms and distinct peaks of both the sample and the internal reference standard. For the samples investigated, the frequency of diploids was 15.2% with a mean genome size of about 775Mb, and tetraploids and hexaploids were 14.4% and 70.3% respectively (Fig. 1, S6 Table). Polyploids, in particular hexaploids, dominate this mixed-ploidy population of *A*. *chinensis*. The morphological differentiation among ploidy levels is shown in Fig. 2A. PC1 and PC2 explained 53.4% and 17.8% of total variance in the data set respectively. PC1 was positively associated with leaf shape (LEL and WIL), while PC2 was negatively associated with the presence/absence of bud cover (PRB) and the size of the hole in the bud cover (SIH) (S2 Table). Both PCs were affected by ploidy level (ANOVA: PC1: *F* = 81.3, *P* < 0.001; PC2: *F* = 10.2, *P* < 0.001). Tukey HSD tests showed that PC1 differed significantly (*P* < 0.001) among the three cytotypes, while only one significant difference, between diploids and hexaploids, was found for PC2 (*P* < 0.001), suggesting the presence of a morphological gradient of tetraploids along this PC. Discriminant analysis revealed that 82% (216/263) of all individuals were correctly assigned into their original ploidy groups based on PCs, and most of the wrong assignments occurred in tetraploids.

Although all three ploidy levels have overlapping niche distribution (Fig. 1), the CCA result reflected the differentiation of cytotype distribution in relation to the geo-environmental variables investigated here (Fig. 2B). The total inertia (the mean squared contingency coefficient) of CCA analysis is 0.753, while the constrained is 0.425, suggesting about 56.44% (0.425/0.753) of the total variation can be explained by variation in nine variables. Permutation test for the whole constrained variables is significant (Pseudo-*F* = 3.454, *P* = 0.001, 999 permutations), suggesting that these variables can largely explain the observed distributing difference between cytotypes, particularly the differentiation between diploids and polyploids (Fig. 2B). The respective significance for each variable were presented in Table 1. The altitude (ALT), niche disturbance (DIS) and vegetation cover (COV) are the most important factors correlating cytotype distribution of *A*. *chinensis* on a local scale. For example, the mean altitude of diploid individuals was 915.4 m a.s.l., which was significantly lower than the mean altitudes of tetraploids (1231.9 m a.s.l.) (*t* = -4.419, *P* < 0.001) and hexaploids (1359.4 m a.s.l.) (*t* = -7.923, *P* < 0.001) that coexisted in this mixed population (Fig. 1).

**Table 1:** The respective significances for each geo-environmental variable examined in the canonical correspondence analysis (CCA) in relation to ploidy distribution of *Actinidia chinensis*

Variable	Abbreviation	CCA1	CCA2	*r* ^2^	*P-*value
Longitude	LON	0.9979	0.0652	0.3701	0.005*
Latitude	LAT	-0.9802	-0.1982	0.4543	0.004*
Altitude	ALT	-0.9971	-0.0760	0.5438	0.001**
Aspect	ASP	0.7736	-0.6337	0.0229	0.719
Slope steepness	SLO	0.4575	0.8892	0.0543	0.431
Soil type	SOI	0.9368	-0.3498	0.0057	0.873
Mesotopography	MES	-0.1144	-0.9934	0.1178	0.155
Niche disturbance	DIS	0.9986	0.0525	0.4047	0.001**
Vegetation cover	COV	0.9994	0.0340	0.3254	0.002*

Significant codes: 0.001, **; 0.01, *. *P* values based on 999 permutations

### Genetic and epigenetic diversity

The eight primer combinations assayed in the AFLP analysis yielded a total of 290 AFLP fragments, of which 88.28% were polymorphic. The five pairs of MSAP primers produced a total of 217 fragments, among which 38 can be classified as nonmethylated loci and 179 as methylation-susceptible loci. Moreover, 65.79% nonmethylated loci and all methylation-susceptible loci are polymorphic. For all methylation-susceptible loci, the methylation rate does not vary much among ploidy levels (29.18%, 27.15% and 28.53% for diploids, tetraploids and hexaploids, respectively). We thus produced a new data set including all of the polymorphic AFLP loci and the nonmethylated loci from MSAP analysis for further genetic analyses. Moreover, the left methylation-susceptible loci from MSAP analysis were considered as really epigenetic data in the following analyses.

As indicated by the Shannon index (*S*), both tetraploids and hexaploids revealed a similar diversity with diploids in both genetic and epigenetic levels (S7 Table). Moreover, mean *S* for genetic loci (0.491±0.018) was similar to that for epigenetic loci (0.389±0.026) (*W* = 322, *P* < 0.001, Wilcoxon rank-sum test) when the comparison was weighted by the number of loci investigated (genetic: *n* = 281; epigenetic: *n* = 179).

### Genetic and epigenetic differentiations

The PCAs of both genetic and epigenetic profiles showed low differentiation among the three ploidy levels (Fig. 2C, D). The PC1 and PC2 explained about 12.4% and 8.0% of the total genetic variations respectively, while the first two PCs explained about 13.5% and 6.5% of epigenetic variation. One-way ANOVA on the first two PCs using ploidy levels as factor revealed that most genetic differentiation occurred in PC2 but not PC1 (PC1: *F* = 0.1, *P* = 0.905; PC2: *F* = 7.2, *P* < 0.001). At the epigenetic level, differentiation was mostly captured by both the first two PCs (PC1: *F* = 3.9, *P* < 0.05; PC2: *F* = 3.2, *P* < 0.05). Tukey HSD tests showed that, for PC2, diploids were genetically different from hexaploids (*P* < 0.001). For epigenetic variation, PC1 separated diploids and hexaploids (*P* < 0.05). In total for all ploidy levels examined, the AMOVAs showed that differentiation among ploidy levels was low both at the genetic (Ф_ST_ = 0.025, *P* < 0.0001) and epigenetic (Ф_ST_ = 0.018, *P* < 0.005) levels (Table 2).

**Table 2:** Multilocus genetic and epigenetic differentiations between *Actinidia chinensis* cytotypes using analyses of molecular variance (AMOVA).

Source of variation		df	Sum of squares	Mean squares	Percentage of variation (%)	*F*-statistics (*P* *)
AFLP	Among cytotypes	2	0.7759	0.3893	2.49	2.41 (*P* < 0.0001)
	Within cytotypes	189	30.3776	0.1603	97.51	
MSAP	Among cytotypes	2	0.4974	0.2487	1.84	1.76 (*P* < 0.005)
	Within cytotypes	189	26.5430	0.1412	98.16	

*P values based on 10 0000 permutations

Mantel test showed a low relationship between the morphological and genetic variation (*r*_M_ = 0.05, *P* < 0.05). The relationship between genotypes and epigenotypes was also weak (*r*_M_ = 0.12, *P* = 0.001). The first three PCs overall were significantly associated to ALT at the epigenetic level (*F* = 78.19, *P* < 0.001, adjusted *R* ^2^ = 0.289) but not at the genetic level (*F* = 20.91, *P* < 0.001, adjusted *R* ^2^ = 0.095). Moreover, nearly no relationship was observed between DIS and molecular variation (genetic: *F* = 4.68, *P* = 0.032, adjusted *R* ^2^ = 0.019; epigenetic: *F* = 20.08, *P* < 0.001, adjusted *R* ^2^ = 0.091).

### Population structure and hybridization

The PPD in STRUCTURE analyses for both genetic and epigenetic data increased from *K* = 1 to *K* = 10, thus the optimal *K* values of both data were determined by the *∆K* (the increase of the PPD). For genetic data, *∆K* is high for *K* = 2, 3 and 4, but greatly decreased when *K* > 4, while for epigenetic data, *∆K* is high for both *K* = 2 and 3, but exhibited decreasing values when *K* > 3 (S1 Fig.). Therefore, we choose the optimal *K* = 4 for genetic data and *K* = 3 for epigenetic data.

**Fig 3:**
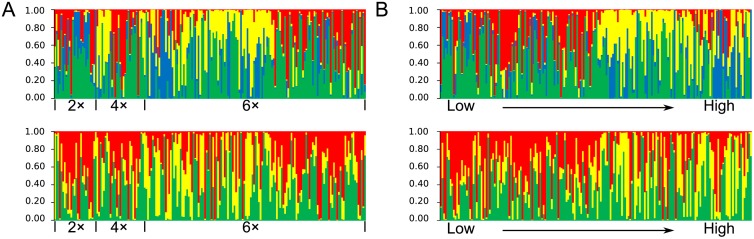
Population genetic and epigenetic structure subdivisions detected in STRUCTURE. (a) *Actinidia chinensis* individuals grouped by ploidy levels and (b) sorted out by sampling sites along altitudinal changes (Low → High). The four different colors in the first row represent four main genetic clusters (*K* = 4, red: cluster I; green: cluster II; yellow: cluster III; blue: cluster IV) and three colors in the second row represent three epigenetic clusters (*K* = 3, red: cluster I; green: cluster II; yellow: cluster III).

The population structure modeled by STRUCTURE analyses were presented in Fig. 3. Based on the optimal *K* = 4 for genetic data, only nine diploids had clearly genetic attributes in both cluster I (proportion of membership: *Q*_I-genetic_ = 0.20) and IV (*Q*_IV-genetic_ = 0.16) when a threshold of *q* = 0.8 [24] were used. Moreover, ten tetraploids were found in cluster I (*Q*_I-genetic_ = 0.14), cluster III (*Q*_III-genetic_ = 0.07) and IV (*Q*_IV-genetic_ = 0.14), while 44 hexaploids could be attributed into four genetic clusters respectively with a higher presence in cluster I (*Q*_I-genetic_ = 0.18). Most individuals examined showed genetic mixture with different extents (Fig. 3A). Similarly, the optimal *K* = 3 for epigenetic data showed that about half of the diploids can be clearly defined in distinctly epigenetic cluster in which most grouped in cluster III (*Q*_III-epigenetic_ = 0.32), and nine tetraploids were split in both cluster I (*Q*_I-epigenetic_ = 0.10) and III (*Q*_III-epigenetic_ = 0.21), while the hexaploids were still split but relatively centered in cluster I (*Q*_I-epigenetic_ = 0.12) and III (*Q*_III-epigenetic_ = 0.11). About 68% individuals showed possibly epigenetic mixture (Fig. 3A). When individuals were sorted out along predominantly eco-geographical variables such as the altitudes from low to high without a consideration of ploidy levels, a trend of continuous genetic variation and a gradual increase of epigenomic heterogeneities of individuals can be easily observed (Fig. 3B).

## Discussion

### Cytotype composition and morphological variation of *A*. *chinensis* at a microgeographical scale

Our study showed that a mixture of three ploidy levels of *A*. *chinensis* individuals can grow sympatrically, even though the hexaploids are dominant (70.3%). Moreover, the distribution of ploidy levels in this population is associated with elevation, and also affected by variables such as niche disturbance and vegetation cover. The presence of three ploidy levels in the DLL population in our study is consistent with previous reports of three major ploidy levels in *A*. *chinensis* complex [23, 27]. We did not find any aneuploid intermediate cytotypes, which may reflect prezygotic reproductive isolation and/or a low fertility and viability of aneuploids in this population [2]. The fact that hexaploids are the most frequent in this mixed population is interesting (Fig. 1). In a relatively large scale investigation of ploidy levels across sixteen populations of *A*. *chinensis* in central-western China, diploids were the most prevalent (64.2%), followed by hexaploids and tetraploids [23]. An investigation of ploidy levels over the whole distribution of the *A*. *chinensis* complex in China showed a similar majority of diploids (57.7%) (W. Huang and Z.Z. Li, unpublished). At a broad geographic scale, a gradual, clinal transition from diploid to hexaploid across elevational and longitudinal gradients is apparent [23]. Previous studies of mixed cytotype populations in central and central-western China further found different ploidy levels (e.g., 2*x-*4*x*, 2*x-*6*x*, 4*x-*6*x* and 4*x-*5*x-*6*x*) with variable ratios dependent on local conditions [25, W. Huang and Z.Z. Li, unpublished]. The transect investigated in this study reflects the range of elevation (500–2000 m) where *A*. *chinensis* is observed in the wild [25]. The predominance of the hexaploids thus suggests a specific response of this cytotype to local environmental conditions [10, 41]. The DLL population studied here is located in the Three Gorges region, which is characterized by abundant geological phenomena (including steep mountains) and abrupt transitions between eco-geographical regions. The dominance of hexaploids may reflect local adaptation or improved competitive ability to these conditions.

The niche divergence between cytotypes supports the hypothesis of niche shifts as a result of the competition between ploidy levels during polyploid formation and establishment in diploid parental populations [6, 42]. However, it is unclear whether this mixed-ploidy population is in a stable or transitional stage. The partially overlapping ecological preferences of cytotypes and the wide ecological amplitude of each observed cytotype (Fig. 1) suggest that altitudinal limit is not the only factor determining their distribution. Indeed, both the niche disturbance and the vegetation cover can have significant impact on the cytotype distribution (Fig. 2B). The protected area of the DLL natural reserve can reduce human impact and thereby improve the restoration of the vegetation, which in turn may favor the spread of hexaploids if their current niche favors less disturbed and diverse vegetation. Furthermore, the origin of polyploidy in *A*. *chinensis* is still unresolved [26]. For tetraploids and hexaploids in the complex, both autopolyploidy and allopolyploidy are possible for the formation of polyploids [26]. Given that the perennial and dioecious nature of kiwifruit plants reinforce reticulate gene flow, the dominant hexaploids in the DLL population could result from crosses between selected members of this complex in a limited frequency, but in turn produce diversely dynamic alterations of genomes in different niche habitats after polyploidization as revealed as the highly admixed genomes of the analyzed samples (Fig. 3). Although not examined in the wild, recent artificial crosses between parents varying in ploidy levels have shown that some combinations only generate offspring with a single ploidy level (such as hexaploids) [C.H. Zhong, unpublished]. Therefore, the higher likelihood of producing surviving hexaploid offspring during polyploidization events may have contributed to the abundance of hexaploids, and hexaploids may have higher fitness in the specific niches examined. If directional selection favors hexaploids in the DLL population, the diploids would eventually be outcompeted due to minority cytotype disadvantage [43].

Taxonomic issues relating to varieties in the *A*. *chinensis* complex have been widely discussed [26]. In our study, the diploids and hexaploids differed morphologically and tetraploids presented intermediate forms for most examined characters. For example, the leaf shape, the presence/absence of bud cover and the size of the hole in the bud cover were positively associated with ploidy levels (Fig. 2A). At a fine spatial scale, though individuals exhibit different ploidy levels, there are gradual changes in observed morphological variation linked with changes in eco-geographical variables (Fig. 2A). Similar patterns of morphological variation in relation to ploidy levels have also been observed when they were examined in kiwifruit cultivars with different ploidy levels [30] or in natural populations across a large spatial scale [23]. The significant morphological differentiation between diploids and hexaploids observed in the present study clearly corresponds to the two taxonomic varieties *Ac* and *Ad* [21]. The tetraploids, however, are intermediate forms of both varieties as evidenced by a high level of wrong assignments in discriminant analysis. In artificial crosses of kiwifruit lineages across ploidy levels, different parent combinations of diploids and hexaploids can easily produce tetraploid hybrids [C.H. Zhong, unpublished]. The low frequency of tetraploid may reflect the competitive disadvantage of tetraploids compared to hexaploids when they occur sympatrically (Fig. 1). The factors influencing the reduced competition ability of the tetraploids remain unclear. The genotypic selection patterns, the parental ploidy [44] and other factors all impact the evolutionary fate of tetraploids at a small geographical scale.

### The population molecular diversity and structure

The level of genetic diversity indicated by the Shannon diversity index (mean *S =* 0.491±0.018, S7 Table) found in this mixed ploidy population is comparable with previous investigations conducted in natural populations of *A*. *chinensis* based on different molecular markers of microsatellite and chloroplast markers [22, 24]. An investigation of microsatellite variation in populations of various *Actinidia* taxa including the *A*. *chinensis* complex revealed a high level of genetic variation and heterozygosity (the mean expected heterozygosity *H*_e_ = 0.839) [24]. Similarly, high mean gene diversity (*H*_T_ = 0.772) was observed in the chloroplast genome of *A*. *chinensis* plants [22]. Kiwifruit plants are perennial and dioecious plants characterized by reticulate natural hybridization and introgression [24]. Hybridization together with polyploidy can generate dynamic changes for both genomic structure and function. The high genetic diversity found here may reflect the substantial structuring variation in genomics, which further supported by the variation of genomic sizes identified within each ploidy levels (S6 Table).

Recent increases in studies on natural epigenetic variation showed the important effects of epigenetic diversity on phenotypic variation and/or environmental adaptation [35, 45, 46]. An investigation of the population structuring of epigenetic variation in the southern Spanish violet *Viola cazorlensis* showed significant relationship of epigenetic differentiation to adaptation of populations [35]. Similarly, by analyzing *Dactylorhiza* species with similar genetic heritage, the authors demonstrated that ecological divergence in sibling allopolyploids is largely the result of adaptation achieved by epigenetic effects [45]. At a microgeographical scale, our study found high polymorphism of methylation-susceptible loci and broad individual differences in multilocus epigenotypes comparable to the high AFLP-based genetic diversity in *A*. *chinensis*, though no significant difference in methylation rates was observed between three ploidy levels (Fig. 2, S7 Table). The epigenetic effects on phenotypic variation and/or environmental adaptation of kiwifruit ploidy individuals investigated here can not be clearly characterized. However, the diverse epigenotypes suggested that the epigenetic alterations, including methylation repatterning, occurred widely in this mixed ploidy population. Drastic epigenetic changes are thus considered to have an important effect on the diversification of kiwifruit plants in natural populations.

Our study found molecular variation, in particular for epigenetic diversity, was related to some predominantly geo-environmental variables such as the altitudes. Moreover, a trend of continuous genetic variation and a gradual increase of epigenomic heterogeneities of individuals from low altitudes to high altitudes can be easily observed (Fig. 3). The factors driving the currently genetic and epigenetic compositions of individuals in relation to microgeographical variables in the *A*. *chinensis* mixed ploidy population is complex. The main evolutionary forces of polyploidy and hybridization have blurred the obvious molecular differentiations among cytotypes at both genetic and epigenetic level as indicated by multilocus AMOVA analyses (Table 2). Moreover, the ecological impact is substantial as indicated by multiple linear regression tests, in which both genetic and epigenetic responses are potentially different. For polyploid individuals in this population, both the parental genomes and subsequent hybridization can commonly program their genomic variation into a gradual pattern along eco-geographical gradient (Fig. 3B). Comparatively, epigenetic response to ecological and evolutionary forces are more rapid and fine [47], which result in a gradual increase of epigenomic heterogeneities (Fig. 3B) when the mountain niche-habitat diversity and ploidy levels increased following ecological gradient as well (Fig. 1). Whether epigenetic variation is hitchhiking along with selected genetic variation or a downstream consequence of that genetic variation in wild populations of *A*. *chinensis* cannot yet be determined, though a weak relationship between genotypes and epigenotypes was detected. Recent studies have provided evidence of the alternative actions of genetic and epigenetic variation to phenotypic evolution and adaptation of plants, such as those reported for the combined genetic and epigenetic effects on adaptation of Spanish violet *Viola cazorlensis* [35] or the independent epigenetic effect driving evolution of *Dactylorhiza* species [45]. Hence, the degree to which epigenetic variation is autonomous from genetic variation may depend on both the species and eco-environment examined.

## Conclusions and Implications

Polyploidization and hybridization is source of extensive genomic diversification in *Actinidia* and may have significant impacts on the morphology and ecological distribution of *A*. *chinensis*. The dominance of hexaploids in this population suggests that polyploidization is involved in local adaptation to specific niches within the existing range rather than with an expansion in the species range, which is dominated overall by diploid [42]. Epigenetic variation may also play a more important role in local adaptive responses than genetic variation, and may be acted upon by selection as it influences phenotypic variation. As an important horticultural plant, our study displayed the evolutionary and ecological significance of polyploid *A*. *chinensis* which can guide the current kiwifruit breeding programs. Exploitation and utilization of natural polyploids are an important direction in term of developing superior cultivars of kiwifruit under global changes.

## Supporting Information

S1 Table Geographical coordinates and ploidy distribution of the 34 sampling sites in the *Actinidia chinensis* mixed-ploidy population. (DOC)

S2 Table Morphological characteristics and canonical loading of principal components analysis (PCA) for *Actinidia chinensis* ploidy individuals in the Dalaoling population. (DOC)

S3 Table Main environmental variables invested in each sampling site of *Actinidia chinensis* mixed-ploidy population. (DOC)

S4 Table Adaptors and pre-selective primers used for AFLP and MASP analyses. (DOC)

S5 Table AFLP and MSAP selective primer combinations. (DOC)

S6 Table The relative fluorescence intensity and the estimated genome size for all *Actinidia chinensis* samples examined by a flow cytometric measurement (FCM). (DOC)

S7 Table The amount of genetic and epigenetic diversity for each cytotype assessed by the Shannon diversity index (*S*). (DOC)

S1 Fig The increase of the posterior probability of the data (PPD) of given *K* in STRUCTURE analyses. (PDF)
