# Supplementary material for: The Microgeographical Patterns of Morphological and Molecular Variation of a Mixed Ploidy Population in the Species Complex Actinidia chinensis
Source: PLoS One. 2015 Feb 6;10(2):e0117596. doi: 10.1371/journal.pone.0117596 (PMC4319829; doi:10.1371/journal.pone.0117596)
Supplement: S1 Table — (DOC) [file pone.0117596.s001.doc]

**Table S1** Geographical coordinates and ploidy distribution of the 34 sampling sites in the *Actinidia chinensis* mixed-ploidy population

| Sampling site | Longitude | Latitude | Altitude (m) | Sample number (ploidy level) | Samples used for molecular analyses (ploidy level) |
| --- | --- | --- | --- | --- | --- |
| 1 | 110.98750ºE | 31.01472ºN | 580 | 6 (2x) | 4 (2x) |
| 2 | 110.97722ºE | 31.01694ºN | 609 | 10 (2x), 1 (6x) | 4 (2x), 1 (6x) |
| 3 | 110.97444ºE | 31.01417ºN | 640 | 2 (4x), 1 (6x) | 2 (4x), 1 (6x) |
| 4 | 110.95778ºE | 31.00389ºN | 782 | 1 (2x), 1 (6x) | 1 (2x), 1 (6x) |
| 5 | 110.95472ºE | 31.00750ºN | 889 | 4 (2x), 2 (4x) | 4 (2x), 1 (4x) |
| 6 | 110.95139ºE | 31.00833ºN | 946 | 3 (2x), 1 (4x), 1 (6x) | 1 (2x), 1 (4x), 1 (6x) |
| 7 | 110.95083ºE | 31.01389ºN | 1009 | 2 (2x), 5 (4x) | 2 (2x), 5 (4x) |
| 8 | 110.95167ºE | 31.01889ºN | 1017 | 4 (2x), 1 (4x), 5 (6x) | 3 (2x), 1 (4x), 5 (6x) |
| 9 | 110.95167ºE | 31.02500ºN | 1058 | 4 (4x), 5 (6x) | 2 (4x), 4 (6x) |
| 10 | 110.95167ºE | 31.02806ºN | 1078 | 1 (2x), 1 (4x), 7 (6x) | 1 (2x), 1 (4x), 7 (6x) |
| 11 | 110.95250ºE | 31.02944ºN | 1085 | 2 (2x), 1 (4x), 5 (6x) | 1 (2x), 1 (4x), 5 (6x) |
| 12 | 110.95278ºE | 31.03361ºN | 1150 | 1 (4x), 6 (6x) | 1 (4x), 4 (6x) |
| 13 | 110.95278ºE | 31.03639ºN | 1206 | 1 (2x), 13 (6x) | 1 (2x), 11 (6x) |
| 14 | 110.95250ºE | 31.03833ºN | 1208 | 1 (4x), 9 (6x) | 1 (4x), 7 (6x) |
| 15 | 110.94944ºE | 31.04389ºN | 1258 | 2 (4x), 12 (6x) | 2 (4x), 8 (6x) |
| 16 | 110.94917ºE | 31.04556ºN | 1272 | 1 (2x), 1 (4x), 5 (6x) | 1 (2x), 1 (4x), 2 (6x) |
| 17 | 110.94611ºE | 31.04972ºN | 1290 | 5 (6x) | 5 (6x) |
| 18 | 110.94806ºE | 31.05194ºN | 1314 | 2 (4x), 10 (6x) | 2 (4x), 9 (6x) |
| 19 | 110.94556ºE | 31.05694ºN | 1360 | 2 (4x), 13 (6x) | 2 (4x), 12 (6x) |
| 20 | 110.94667ºE | 31.05778ºN | 1390 | 9 (6x) | 5 (6x) |
| 21 | 110.94639ºE | 31.05889ºN | 1400 | 1 (4x), 5 (6x) | 2 (6x) |
| 22 | 110.94472ºE | 31.06000ºN | 1410 | 2 (2x), 6 (6x) | 1 (2x), 2 (6x) |
| 23 | 110.94306ºE | 31.06333ºN | 1447 | 1 (4x), 7 (6x) | 1 (4x), 3 (6x) |
| 24 | 110.93917ºE | 31.06333ºN | 1468 | 9 (6x) | 5 (6x) |
| 25 | 110.93778ºE | 31.06222ºN | 1485 | 3 (4x), 4 (6x) | 1 (4x), 2 (6x) |
| 26 | 110.92139ºE | 31.08611ºN | 1558 | 2 (2x), 11 (6x) | 1 (2x), 7 (6x) |
| 27 | 110.92778ºE | 31.06278ºN | 1579 | 1 (2x), 5 (6x) | 4 (6x) |
| 28 | 110.92028ºE | 31.06806ºN | 1655 | 2 (6x) | 2 (6x) |
| 29 | 110.92222ºE | 31.07861ºN | 1667 | 1 (2x), 2 (4x), 10 (6x) | 1 (2x), 1 (4x), 7 (6x) |
| 30 | 110.92389ºE | 31.07250ºN | 1706 | 2 (6x) | 2 (6x) |
| 31 | 110.92361ºE | 31.07611ºN | 1721 | 1 (2x), 5 (6x) | 1 (2x), 5 (6x) |
| 32 | 110.93028ºE | 31.07222ºN | 1753 | 5 (6x) | 2 (6x) |
| 33 | 110.92972ºE | 31.07333ºN | 1754 | 1 (4x), 4 (6x) | 1 (4x), 4 (6x) |
| 34 | 110.93833ºE | 31.07889ºN | 1826 | 2 (4x), 2 (6x) | 1 (4x), 2 (6x) |
