# Supplementary material for: The Microgeographical Patterns of Morphological and Molecular Variation of a Mixed Ploidy Population in the Species Complex Actinidia chinensis
Source: PLoS One. 2015 Feb 6;10(2):e0117596. doi: 10.1371/journal.pone.0117596 (PMC4319829; doi:10.1371/journal.pone.0117596)
Supplement: S2 Table — (DOC) [file pone.0117596.s002.doc]

Table S2 Morphological characteristics and canonical loading of principal components analysis (PCA) for *Actinidia chinensis* ploidy individuals in the Dalaoling population

| Characteristic | Abbreviation | PC1 | PC2 |
| --- | --- | --- | --- |
| 1. Diameter of stem (mm) | DOS | 0.09135476 | 0.09622470 |
| 2. Length of petiole (cm) | LEP | 0.26265071 | -0.34913551 |
| 3. Length of leaf blade (cm) | LEL | **0.50608812** | -0.11709109 |
| 4. Diameter of petiole (mm) | DIP | 0.29466400 | -0.20010767 |
| 5. Width of leaf blade (cm) | WIL | **0.47035631** | -0.21890623 |
| 6. Length of stem (cm) | LES | 0.33615358 | -0.02568740 |
| 7. Size of hole in bud cover (small, 0; medium, 1; large, 2) | SIH | -0.17850276 | **-0.52884159** |
| 8. Size of bud support of stem (small, 0; medium, 1; large, 2) | SIB | 0.23153381 | 0.10152517 |
| 9. Shape of leaf blade (broad obovate, 0; ovate, 1; lanceolate, 2) | SHB | -0.01557979 | 0.04378446 |
| 10. Texture of leaf blade (papery, 0; thick papery, 1; leather, 2) | TEL | -0.17757104 | -0.19914338 |
| 11. Type of hairiness of young shoot (downy, 0; tomentose, 1) | TYH | 0.32473879 | 0.37881806 |
| 12. Size of lenticels of stem (small, 0; medium, 1; large, 2) | SIL | -0.03458078 | -0.11260203 |
| 13. Shape of lenticels of stem (ellipse, 0; shuttle, 1) | SHL | -0.09654420 | -0.00918322 |
| 14. Shape of apex of leaf blade (rounded and emarginated, 0; caudate, 1; acuminate, 2) | SHA | 0.05052480 | 0.01284313 |
| 15. Presence of bud cover (absent, 0; present, 1) | PRB | -0.01977512 | **-0.47443933** |
| 16. Arrangement of leaf basal lobes (apart, 0; overlapping, 1) | ARB | 0.09654803 | 0.23115315 |

PC1 and PC2 indicate the first and second principal components of PCA. Bold type indicates the dominant functions.
