# Supplementary material for: The Microgeographical Patterns of Morphological and Molecular Variation of a Mixed Ploidy Population in the Species Complex Actinidia chinensis
Source: PLoS One. 2015 Feb 6;10(2):e0117596. doi: 10.1371/journal.pone.0117596 (PMC4319829; doi:10.1371/journal.pone.0117596)
Supplement: S3 Table — (DOC) [file pone.0117596.s003.doc]

Table S3 Main environmental variables invested in each sampling site of *Actinidia chinensis* mixed-ploidy population

| Variable | Status descriptor |
| --- | --- |
| Slope steepness | <5º (1), 15-30º (2), 30-45º (3), 45-60º (4), >60º (5) |
| Aspect | N (1), NE (2), E(3), SE (4), S (5), SW (6), W (7), NW (8) |
| Mesotopography | Crest (1), Upper slope (2), Mid slope (3), Lower slope (4), Gully (5) |
| Soil type | Red earths (1), Yellow earths (2), mountain yellow-brown earths (3), mountain brown earths (4) |
| Niche disturbance | Stable (1), Nearly stable (2), Unstable (3), Strongly unstable (4) |
| Vegetation cover | <5% (1), 5-10% (2), 10-25% (3), 25-50% (4), 50-75% (5), >75% (6) |
