# Supplementary material for: The Microgeographical Patterns of Morphological and Molecular Variation of a Mixed Ploidy Population in the Species Complex Actinidia chinensis
Source: PLoS One. 2015 Feb 6;10(2):e0117596. doi: 10.1371/journal.pone.0117596 (PMC4319829; doi:10.1371/journal.pone.0117596)
Supplement: S4 Table — (DOC) [file pone.0117596.s004.doc]

Table S4 Adaptors and pre-selective primers used for AFLP and MASP analyses

| Adapter | *Mse*I-adapter I | 5′-GACGATGAGTCCTGAG |
| --- | --- | --- |
|  | *Mse*I-adapter II | 5′-TACTCAGGACTCAT |
|  | *EcoR*I-adapter I | 5′-CTCGTAGACTGCGTACC |
|  | *EcoR*I-adapter II | 5′-AATTGGTACGCAGTC |
|  | HM-adapter I | 5′-GATCATGAGTCCTGCT |
|  | HM-adapter I | 5′-CGAGCAGGACTCATGA |
| Pre-selective primer | *Mse*I + C | 5′-GACGATGAGTCCTGAGTAAC |
|  | *EcoR*I + A | 5′-GACTGCGTACCAATTCA |
|  | HM + T | 5′-ATCATGAGTCCTGCTCGGT |
