# Supplementary material for: The Microgeographical Patterns of Morphological and Molecular Variation of a Mixed Ploidy Population in the Species Complex Actinidia chinensis
Source: PLoS One. 2015 Feb 6;10(2):e0117596. doi: 10.1371/journal.pone.0117596 (PMC4319829; doi:10.1371/journal.pone.0117596)
Supplement: S5 Table — (DOC) [file pone.0117596.s005.doc]

Table S5 AFLP and MSAP selective primer combinations

| Labelled primer | Unlabelled primer | | | | | | | | |
| --- | --- | --- | --- | --- | --- | --- | --- | --- | --- |
|  | Mse-CAC | Mse-CTA | Mse-CTG | Mse-ctt | Mse-CAT | HM-TCC | HM-TCA | HM-TTC | HM-TGA |
| E-AGC | AFLP#1 | - | AFLP#3 | - | - | MSAP#1 | - |  | MSAP#5 |
| E-ACC | - | AFLP#2 | - | - | - | - | MSAP#2 | - | - |
| E-ACT | - | - | - | - | AFLP#5 | - | - | - | - |
| E-AGG | AFLP#8 | AFLP#6 | - | AFLP#4 | - | - | - | MSAP#3 | - |
| E-ACA | - | - | - | - | - | - | - | MSAP#4 | - |
| E-ATC | - | - | - | AFLP#7 | - | - | - | - | - |
