# Supplementary material for: The Microgeographical Patterns of Morphological and Molecular Variation of a Mixed Ploidy Population in the Species Complex Actinidia chinensis
Source: PLoS One. 2015 Feb 6;10(2):e0117596. doi: 10.1371/journal.pone.0117596 (PMC4319829; doi:10.1371/journal.pone.0117596)
Supplement: S6 Table — (DOC) [file pone.0117596.s006.doc]

**Table S6** The relative fluorescence intensity and the estimated genome size for all *Actinidia chinensis* samples examined by a flow cytometric measurement (FCM)

| Samples | Relative fluorescence intensity | Estimated genome size |
| --- | --- | --- |
| 1 | 104.06 | 1560.9 |
| 2 | 103.44 | 1551.6 |
| 3 | 156.92 | 2353.8 |
| 4 | 147.49 | 2212.35 |
| 5 | 151.08 | 2266.2 |
| 6 | 104.46 | 1566.9 |
| 7 | 157.59 | 2363.85 |
| 8 | 148.78 | 2231.7 |
| 9 | 151.23 | 2268.45 |
| 10 | 155.1 | 2326.5 |
| 11 | 150.56 | 2258.4 |
| 12 | 141.63 | 2124.45 |
| 13 | 150.66 | 2259.9 |
| 14 | 147.51 | 2212.65 |
| 15 | 145.97 | 2189.55 |
| 16 | 152.01 | 2280.15 |
| 17 | 151.36 | 2270.4 |
| 18 | 145.84 | 2187.6 |
| 19 | 143.29 | 2149.35 |
| 20 | 49.15 | 737.25 |
| 22 | 153.57 | 2303.55 |
| 24 | 149.24 | 2238.6 |
| 26 | 144.45 | 2166.75 |
| 27 | 136.18 | 2042.7 |
| 28 | 147.8 | 2217 |
| 29 | 144.23 | 2163.45 |
| 30 | 157.47 | 2362.05 |
| 31 | 138.21 | 2073.15 |
| 32 | 99.64 | 1494.6 |
| 33 | 102 | 1530 |
| 34 | 145.34 | 2180.1 |
| 35 | 150.08 | 2251.2 |
| 36 | 56.3 | 844.5 |
| 37 | 164.54 | 2468.1 |
| 38 | 151.4 | 2271 |
| 39 | 60.71 | 910.65 |
| 40 | 149.06 | 2235.9 |
| 41 | 157.32 | 2359.8 |
| 42 | 148.4 | 2226 |
| 43 | 143.69 | 2155.35 |
| 44 | 144.54 | 2168.1 |
| 45 | 146.2 | 2193 |
| 46 | 144.01 | 2160.15 |
| 47 | 149.25 | 2238.75 |
| 48 | 50.68 | 760.2 |
| 49 | 148.06 | 2220.9 |
| 50 | 153.63 | 2304.45 |
| 51 | 148.09 | 2221.35 |
| 52 | 147.27 | 2209.05 |
| 53 | 146.88 | 2203.2 |
| 54 | 141.05 | 2115.75 |
| 55 | 143.52 | 2152.8 |
| 56 | 154.7 | 2320.5 |
| 57 | 50.51 | 757.65 |
| 58 | 144.01 | 2160.15 |
| 59 | 145.06 | 2175.9 |
| 60 | 150.93 | 2263.95 |
| 61 | 149.31 | 2239.65 |
| 62 | 102.1 | 1531.5 |
| 63 | 97.17 | 1457.55 |
| 64 | 151.02 | 2265.3 |
| 65 | 156.11 | 2341.65 |
| 66 | 102.71 | 1540.65 |
| 67 | 149.91 | 2248.65 |
| 68 | 146.67 | 2200.05 |
| 69 | 147.51 | 2212.65 |
| 70 | 145.97 | 2189.55 |
| 71 | 151.21 | 2268.15 |
| 72 | 148.74 | 2231.1 |
| 73 | 150.08 | 2251.2 |
| 74 | 142.79 | 2141.85 |
| 75 | 143.29 | 2149.35 |
| 76 | 150.41 | 2256.15 |
| 77 | 107.31 | 1609.65 |
| 78 | 147.11 | 2206.65 |
| 79 | 154.37 | 2315.55 |
| 80 | 150.72 | 2260.8 |
| 81 | 147.99 | 2219.85 |
| 82 | 149.76 | 2246.4 |
| 83 | 139.98 | 2099.7 |
| 84 | 98.93 | 1483.95 |
| 85 | 104.26 | 1563.9 |
| 86 | 158.81 | 2382.15 |
| 87 | 155.76 | 2336.4 |
| 88 | 151.54 | 2273.1 |
| 89 | 152.23 | 2283.45 |
| 90 | 160.95 | 2414.25 |
| 91 | 153.12 | 2296.8 |
| 92 | 150.91 | 2263.65 |
| 93 | 147.88 | 2218.2 |
| 94 | 108.06 | 1620.9 |
| 95 | 153.43 | 2301.45 |
| 96 | 146.74 | 2201.1 |
| 97 | 143.31 | 2149.65 |
| 98 | 141.87 | 2128.05 |
| 99 | 159.29 | 2389.35 |
| 100 | 152.67 | 2290.05 |
| 101 | 150.87 | 2263.05 |
| 102 | 145.67 | 2185.05 |
| 103 | 139.98 | 2099.7 |
| 104 | 147.78 | 2216.7 |
| 105 | 160.34 | 2405.1 |
| 106 | 152.76 | 2291.4 |
| 107 | 149.2 | 2238 |
| 108 | 145.98 | 2189.7 |
| 109 | 104.06 | 1560.9 |
| 110 | 155.73 | 2335.95 |
| 111 | 96.46 | 1446.9 |
| 112 | 147.28 | 2209.2 |
| 113 | 142.94 | 2144.1 |
| 114 | 148.65 | 2229.75 |
| 115 | 153.57 | 2303.55 |
| 116 | 157.07 | 2356.05 |
| 117 | 153.77 | 2306.55 |
| 118 | 154.95 | 2324.25 |
| 119 | 145.47 | 2182.05 |
| 120 | 157.38 | 2360.7 |
| 121 | 144.31 | 2164.65 |
| 122 | 156.88 | 2353.2 |
| 123 | 149.81 | 2247.15 |
| 124 | 146.86 | 2202.9 |
| 125 | 149.98 | 2249.7 |
| 126 | 108.91 | 1633.65 |
| 127 | 145.27 | 2179.05 |
| 128 | 147.96 | 2219.4 |
| 129 | 152.34 | 2285.1 |
| 130 | 94.36 | 1415.4 |
| 131 | 144.54 | 2168.1 |
| 132 | 155.94 | 2339.1 |
| 133 | 153.19 | 2297.85 |
| 134 | 149.42 | 2241.3 |
| 135 | 161.28 | 2419.2 |
| 136 | 147.79 | 2216.85 |
| 137 | 161.57 | 2423.55 |
| 138 | 159.67 | 2395.05 |
| 139 | 144.12 | 2161.8 |
| 140 | 140.73 | 2110.95 |
| 141 | 157.55 | 2363.25 |
| 142 | 159.15 | 2387.25 |
| 143 | 47.5 | 712.5 |
| 144 | 162.81 | 2442.15 |
| 145 | 110.33 | 1654.95 |
| 146 | 105 | 1575 |
| 147 | 98.7 | 1480.5 |
| 148 | 159.25 | 2388.75 |
| 149 | 140.03 | 2100.45 |
| 150 | 160.34 | 2405.1 |
| 151 | 157.95 | 2369.25 |
| 152 | 160.23 | 2403.45 |
| 153 | 152.72 | 2290.8 |
| 154 | 156.29 | 2344.35 |
| 155 | 153.07 | 2296.05 |
| 156 | 159.07 | 2386.05 |
| 157 | 155.31 | 2329.65 |
| 158 | 157.23 | 2358.45 |
| 159 | 152.85 | 2292.75 |
| 160 | 160.58 | 2408.7 |
| 161 | 162.66 | 2439.9 |
| 162 | 147.8 | 2217 |
| 163 | 155.78 | 2336.7 |
| 164 | 156.86 | 2352.9 |
| 165 | 164.36 | 2465.4 |
| 166 | 158.19 | 2372.85 |
| 167 | 102.43 | 1536.45 |
| 168 | 163.54 | 2453.1 |
| 169 | 158.16 | 2372.4 |
| 170 | 151.48 | 2272.2 |
| 171 | 150.7 | 2260.5 |
| 172 | 138.31 | 2074.65 |
| 173 | 136.18 | 2042.7 |
| 174 | 163.55 | 2453.25 |
| 175 | 147.71 | 2215.65 |
| 176 | 151.03 | 2265.45 |
| 177 | 50.56 | 758.4 |
| 178 | 155.29 | 2329.35 |
| 179 | 141.07 | 2116.05 |
| 180 | 147.36 | 2210.4 |
| 181 | 148.45 | 2226.75 |
| 182 | 141.16 | 2117.4 |
| 183 | 151.62 | 2274.3 |
| 184 | 148.77 | 2231.55 |
| 185 | 100.79 | 1511.85 |
| 186 | 149.63 | 2244.45 |
| 187 | 143.99 | 2159.85 |
| 188 | 147.92 | 2218.8 |
| 189 | 145.48 | 2182.2 |
| 190 | 149.91 | 2248.65 |
| 191 | 112.76 | 1691.4 |
| 192 | 150 | 2250 |
| 193 | 56.07 | 841.05 |
| 194 | 48.19 | 722.85 |
| 195 | 148.98 | 2234.7 |
| 196 | 159.75 | 2396.25 |
| 197 | 146.95 | 2204.25 |
| 198 | 151.35 | 2270.25 |
| 199 | 54.04 | 810.6 |
| 200 | 147.69 | 2215.35 |
| 201 | 103.67 | 1555.05 |
| 202 | 149.41 | 2241.15 |
| 203 | 152.51 | 2287.65 |
| 204 | 142.88 | 2143.2 |
| 205 | 147.82 | 2217.3 |
| 206 | 153.84 | 2307.6 |
| 207 | 153.44 | 2301.6 |
| 208 | 146.66 | 2199.9 |
| 209 | 150.9 | 2263.5 |
| 210 | 99.66 | 1494.9 |
| 211 | 145.03 | 2175.45 |
| 212 | 101.78 | 1526.7 |
| 213 | 113.21 | 1698.15 |
| 214 | 149.34 | 2240.1 |
| 215 | 143.13 | 2146.95 |
| 216 | 92.68 | 1390.2 |
| 217 | 51.48 | 772.2 |
| 218 | 55.62 | 834.3 |
| 219 | 54.93 | 823.95 |
| 220 | 56.11 | 841.65 |
| 221 | 98.23 | 1473.45 |
| 222 | 145.97 | 2189.55 |
| 223 | 142.94 | 2144.1 |
| 224 | 142.44 | 2136.6 |
| 225 | 149.35 | 2240.25 |
| 226 | 146.67 | 2200.05 |
| 227 | 104.87 | 1573.05 |
| 228 | 106.21 | 1593.15 |
| 230 | 141.81 | 2127.15 |
| 231 | 45.68 | 685.2 |
| 232 | 48.93 | 733.95 |
| 233 | 56.08 | 841.2 |
| 234 | 144.37 | 2165.55 |
| 235 | 56.53 | 847.95 |
| 236 | 51.33 | 769.95 |
| 237 | 53.08 | 796.2 |
| 238 | 45.39 | 680.85 |
| 239 | 47.16 | 707.4 |
| 240 | 53.55 | 803.25 |
| 241 | 49 | 735 |
| 244 | 49.08 | 736.2 |
| 245 | 49.56 | 743.4 |
| 246 | 47.41 | 711.15 |
| 247 | 49.57 | 743.55 |
| 248 | 52.06 | 780.9 |
| 249 | 50.63 | 759.45 |
| 251 | 153.99 | 2309.85 |
| 253 | 47.65 | 714.75 |
| 254 | 49.41 | 741.15 |
| 255 | 50.98 | 764.7 |
| 257 | 48.25 | 723.75 |
| 258 | 42.21 | 633.15 |
| 259 | 96.34 | 1445.1 |
| 260 | 107.2 | 1608 |
| 261 | 43.48 | 652.2 |
| 262 | 105.34 | 1580.1 |
| 263 | 141.12 | 2116.8 |
| 264 | 47.33 | 709.95 |
| 265 | 48.93 | 733.95 |
| 266 | 99.8 | 1497 |
| 267 | 96.34 | 1445.1 |
| 268 | 90.79 | 1361.85 |
| 269 | 113.6 | 1704 |
| 270 | 46 | 690 |
| 271 | 108.98 | 1634.7 |
| 272 | 51.56 | 773.4 |
