# Supplementary material for: The Microgeographical Patterns of Morphological and Molecular Variation of a Mixed Ploidy Population in the Species Complex Actinidia chinensis
Source: PLoS One. 2015 Feb 6;10(2):e0117596. doi: 10.1371/journal.pone.0117596 (PMC4319829; doi:10.1371/journal.pone.0117596)
Supplement: S7 Table — (DOC) [file pone.0117596.s007.doc]

**Table S7** The amount of genetic and epigenetic diversity for each cytotype assessed by the Shannon diversity index (*S*)

| Ploidy | *S-*_genetic_ | *S-*_epigenetic_ |
| --- | --- | --- |
| Diploid | 0.485±0.017 | 0.393±0.023 |
| Tetraploid | 0.491±0.028 | 0.386±0.022 |
| Hexaploid | 0.492±0.029 | 0.390±0.027 |
| Mean | 0.491±0.018 | 0.389±0.026 |
