# Supplementary material for: The Microgeographical Patterns of Morphological and Molecular Variation of a Mixed Ploidy Population in the Species Complex Actinidia chinensis
Source: PLoS One. 2015 Feb 6;10(2):e0117596. doi: 10.1371/journal.pone.0117596 (PMC4319829; doi:10.1371/journal.pone.0117596)
Supplement: S1 Fig — (PDF) [file pone.0117596.s008.pdf]

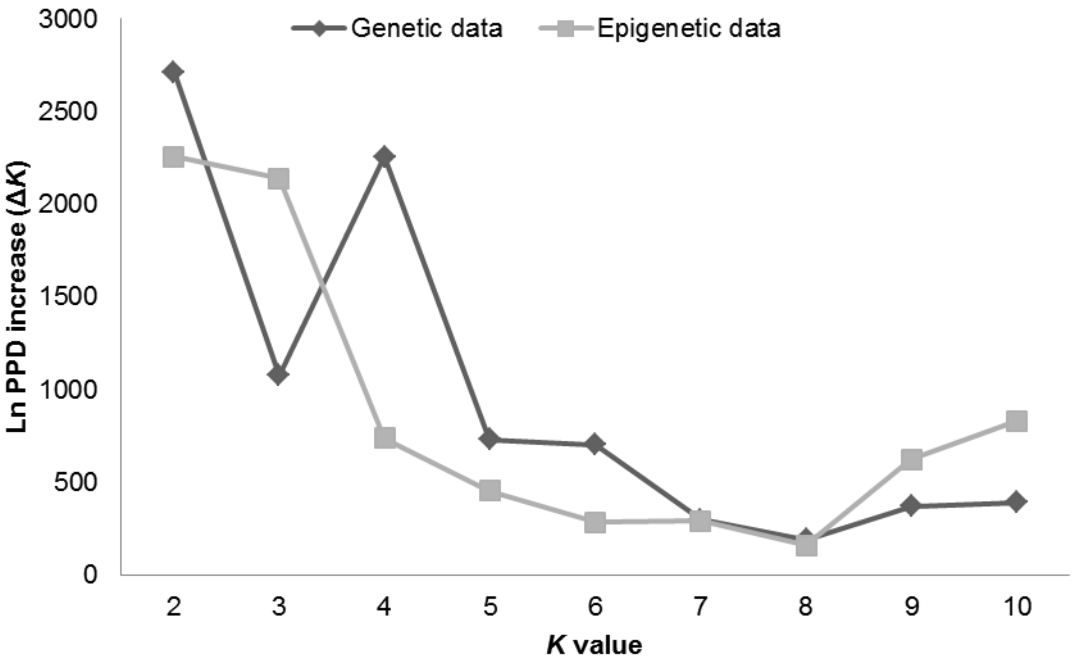

Figure S1 The increase of the posterior probability of the data (PPD) of given *K* in STRUCTURE analyses. For *K* values from 1 to 10, this increase is calculated as $\Delta K = \text{Ln PPD}_K - \text{Ln PPD}_{K\text{-}1}$.
